# Professor Sims Woodhead at the Hospital Saturday Fund

**Published:** 1906-05-05

**Authors:** 


### PROFESSOR SIMS WOODHEAD AT THE HOSPITAL SATURDAY FUND.

### AN ABSTRACT OF THE ADDRESS.

Many factors play a part in determining the public health, and of all these, good nutrition, using the term in its widest sense, is probably the most important. It is necessary that we should have some knowledge of the phenomena of nutrition of the cells of the body, and of the conditions under which healthy nutrition can be carried on in them. We have to consider also how far healthy function is associated with healthy nutrition of these cells, and how the waste products formed during the building up of the tissues and the conversion of food into energy are thrown out, or, when stored up in the cell, act to its detriment. It is essential, of course, that sufficient proper food should, in the first instance, be taken into the body by digestion in the alimentary canal and that soluble nutritive substances prepared should be conveyed by the blood-vessels and lymphatics to the various tissues which are built up, and maintained in their doing effective work. It is not enough, however, that the food should be sufficient. The cells or tissue elements must be sufficiently active to take in and digest this food, to utilise it properly, and to throw out all waste material as it is formed. No tissue of the body can be maintained in an active, healthy condition, unless it is kept doing work. If the muscles of the arm are to be developed properly, they must be exercised; and only so can the brain and the other organs of the body be kept sound. It is fully recognised by pathologists that wasting of an organ may occur as the result (1) of imperfect nutrition, due to want of food; or (2) of imperfect nutrition, due to want of exercise. In addition to this there is (3) a form which appears to be brought about by overwork, especially if long continued, even when the amount of proper nutriment supplied appears to be sufficient. This third form appears to ensue because the tissues are not afforded a long enough period of rest to enable them to make good the waste involved when the work is done, or to permit of the tissue getting rid of the effete matter which results from this same activity. There is going on in the body a constant process of building up and elimination of waste products. These waste products are, in many cases, intensely toxic, and interfere with the activity of the cells. Beyond all these—food and exercise, rest and repair—it is essential that no poisonous substances, whether formed within or outside the body, should be allowed to remain in contact with healthy cell substance for any prolonged period. Now, if individuals are to be maintained at their highest efficiency as workers, it will be evident that the question becomes immensely more important when we consider it in relation to the community as a whole, for we must recognise that if we are to maintain the public health at the highest pitch of perfection, the state of health and of powers of doing work of individuals must be carefully studied, and steps taken to counteract any and all of the conditions that affect them adversely.

#### Better Value for Money Expended.

Just as we are beginning to realise that we cannot legislate in compartments, so we cannot study this question of nutrition without bringing in factors which hitherto have received but little consideration. I am thoroughly satisfied, from a careful study of statistics, that the amount of alcohol consumed amongst certain classes is *the* factor which determines whether a family shall be properly or improperly nourished; that is, whether a certain wage can be looked upon as being a living wage or not. Dr. Dawson Burns and Mr. T. P. Whittaker have enabled us to calculate that £18 a year of a working man's wage is spent on alcohol. Of course, this sum of £18 is greatly exceeded in some families, whilst less is so spent in others, but we may take it that a shilling a day, Saturday, Sunday, and throughout the week, may be taken as a fair average expenditure on this item. Allowing all that is claimed by certain experimenters for alcoholic beverages as food material (taking Dr. Hutchinson's figures) only one-eighth of the amount of nutriment can be obtained from the cheapest forms that can be derived from a similar sum expended on bread, also of the cheapest kind. We may say, therefore, that with this expenditure on alcohol, there is, from the point of view of nutrition, a dead loss of about six shillings a week. This six shillings spent on bread, or even on meat, dried fish, peas, or other nutritive material, would add enormously to the nutritive value of the diet of the family, and not only would the wage earner be better nourished—and he, of course, must be as fully nourished as possible, if he is to be fully effective—but there would be a considerable balance left over for the building up of the physique of the mother who gives birth to the children, and for the proper nutrition of the young and growing members of the family. It may be objected that many of those who spend this six or seven shillings a week on alcohol have still sufficient to provide enough food for the family; but, again, it may be pointed out that even then more clothing and better housing could be provided than is at present obtainable.

#### Effects on Infant Mortality.

There is one field in public health in which the part played by alcohol is only now coming to be fully realised. Although there has been such a marked fall in the general death-rate—and in some cities the death-rate has fallen by one-half during the last thirty or thirty-five years—the infant mortality does not show a corresponding fall. Unfortunately, in our large towns the wife is copying her husband in the matter of alcoholic excess, and impairment of nutrition, so important to the wage-earner, is even more important in the case of the mother and of her unborn child which, as a result of malnutrition during the early stages of its development, comes into the world terribly handicapped, for under the most favourable circumstances it has but a shortened life to live. What I wish to bring out from all this is, that the effects on the public health of the alcohol consumed are both direct and indirect, and that every million we can knock off our drink bill, though not all gain, will certainly make for an improved public health.

#### Alcohol and Disease.

Now, let us go a step further, and see how the consumption of alcohol affects (1) the spread of disease, and (2) the attacks of disease on the individual. In our large cities, as Dr. Niven has indicated, the spread of consumption or tuberculosis is brought about in various ways. He refers especially to the large part the public-houses play in this connection. He points out that people of all sorts and conditions, and in various stages of consumption, meet; some in advanced stages of the disease spread the virus of the disease in a most careless and indiscriminate fashion by expectorating in crowded and ill-ventilated rooms, in which, of course, there is a constant stirring up of what falls to the floor. These people herding together are, many of them, prepared, by lack of nourishing food and by intemperance, for ready development of the disease on the reception of infective material: everything seems to favour its spread. In France, the association of cause and effect has been so carefully observed that it is now stated most confidently that the deaths from consumption in any definite area vary directly with the amount of alcohol consumed.

### Phagocytes and Opsonins.

Experiments carried out in various pathological laboratories afford an additional weight of evidence on one of these points, as it has been found that animals treated with alcohol are much more susceptible to infection by the tubercle bacillus than are animals not so treated; whilst, as regards the result of clinical experience, we have only to refer to the best medical text-books to learn that the consensus of medical opinion is to the effect that alcoholism is one of the great (if not the greatest) predisposing factors in the etiology of tuberculosis. What is true of tuberculosis is true of many other infective diseases, concerning which definite data have been obtained. Against some of these infective diseases it has been shown that it is almost impossible to obtain a perfect immunity in alcoholised animals, although in non-alcoholised animals such immunity may be very readily produced. All this, of course, has a very marked bearing on the prevention of disease, as also on its cure, for, if alcohol interferes with the production of immunity, it must necessarily interfere with cure, as in most of these specific infective diseases cure only takes place where the tissues are able to acquire an immunity or resisting power, temporary or permanent, against the causal agent of the disease. Bearing on this point, Professor A. E. Wright has been able to show that under certain conditions and in certain diseases there is a production, in the bodies of patients, of substances—a special one for each disease—which, acting upon the bacilli associated with the disease, prepare or "cook" them in order that the "phagocytes," or devouring white corpuscles that circulate in the blood, may eat them up readily and quickly. He finds that by appropriate treatment he is able to increase the amount of these specific substances, or "opsonins" as he calls them (because they cook or prepare the bacilli for the phagocytes). He has observed, especially in tuberculosis, that even cases in which these opsonins appear to be readily formed when no alcohol is given to the patient, seem to lose their power of producing these substances when alcohol is given, and, as these opsonins appear to be associated with the cure of the disease, the falling-off in their production, brought about by the use of alcohol, must necessarily mean less favourable conditions for the cure of patients. The more we learn concerning alcohol and its effects on the body, the more fully we recognise that most of these effects are harmful. The importance of the alcohol question to our hospitals can scarcely be over-estimated, as in both medical and surgical cases—*i.e.* as a factor in the production of disease, and in the causation of accidents, it plays a prominent part, whilst in rendering the patient unable to bear operations or to acquire immunity, its *role* is almost overwhelming.

### Insurance Mortality Experience.

I should like to say a few words about a new method of treating insurance statistics, which has just been put forward by Dr. Neil Carmichael, of Glasgow. This method is specially valuable, because it brings out the alcohol factor in disease, accidents, and suicide more clearly than has hitherto been done. From the public health and hospital point of view, it is, therefore, specially instructive. We have, of course, for long been aware that insurance companies, not by any means sentimental corporations, have a special affection for total abstainers. The reasons for this we have in the following statistics from the Forty-first Annual Report of the Sceptre Life Association. In the "general" section the percentage of actual deaths to those expected under the Healthy Males Table last year was 79.58, while in the "Temperance" section it was only 48.31. For the past twenty-two years the percentages were 79.53 and 54.25 respectively. It may be objected that although on the whole total abstainers may live a little longer than moderate drinkers, total abstainers must suffer more from certain diseases and die from certain diseases, and at a greater rate even than moderate drinkers. Dr. Carmichael supplies the most conclusive answer to this objection. He first makes a preliminary analysis of a series of figures collected from a temperance assurance association for the twenty years 1883-1902, calculating the claims expected in each section, temperance and general, on the Healthy Males Table, and these show a difference of 26 per cent. in favour of the temperance section. Taking these figures as his working material, and adopting the headings of the Registrar-General, Dr. Carmichael shows that under every one of these headings there is a greater mortality amongst the moderate drinkers (and, of course, none but moderate drinkers are admitted for insurance at ordinary rates, or indeed at any rate) than amongst abstainers. He says "the deaths in the non-abstainers' section are in excess of those in the abstainers' section in all classes of disease. The excess is not limited to one class of disease, and is not counterbalanced by an excess in any class of disease in the abstainers' section. It is recognised that excessive drinking tends to produce certain diseases, and the Registrar-General has shown in his annual report for 1897 that in certain trades associated with the manufacture or sale of alcohol in its various forms the mortality from certain diseases is very great. Amongst those diseases he places—diseases of the nervous system, diseases of the liver, influenza, rheumatic fever, and diseases of the heart and circulatory system. We find that the excess of mortality in the non-abstainers' section from these diseases is very considerable. In diseases of the nervous system the excess is 150 per cent.; in diseases of the liver it is 194 per cent.; in influenza, 194 per cent.; in rheumatic fever, 284 per cent.; and in diseases of the heart and circulatory system, 52 per cent." Even under the other headings, although the excess of mortality is not so great, it is still appreciable, and we may sum up the result of these investigations in the statement that the mortality amongst total abstainers is greater in no single class or group of causes of death but one, and that one—the only cause from which total abstainers die at a greater rate than their "moderate" brethren—is real old age.

